# A Core Outcome Set for nutritional intervention studies in older adults with malnutrition and those at risk: a study protocol

**DOI:** 10.1186/s12877-023-03832-2

**Published:** 2023-04-06

**Authors:** Marjolein Visser, Nuno Mendonça, Christina Avgerinou, Sibel Cavdar, Tommy Cederholm, Alfonso J. Cruz-Jentoft, Eva Kiesswetter, Hanna M. Siebentritt, Cornel Sieber, Gabriel Torbahn, Dorothee Volkert

**Affiliations:** 1 Department of Health Sciences, Faculty of Science, Vrije Universiteit Amsterdam, Amsterdam Public Health Research Institute, De Boelelaan 1085, Amsterdam, 1081 HV the Netherlands; 2 EpiDoC Unit, CEDOC, NOVA Medical School, Universidade Nova de Lisboa, Rua do Instituto Bacteriológico n°5, Lisbon, 1150-082 Portugal; 3 Comprehensive Health Research Centre (CHRC), NOVA Medical School, Universidade Nova de Lisboa, Lisbon, Portugal; 4 Research Department of Primary Care and Population Health, University College London, London, UK; 5 Division of Geriatrics, Department of Internal Medicine, Ege University Hospital, Izmir, Turkey; 6 Ege University Hospital, Kazımdirik, University Street. No:9, Bornova/İzmir, 35100 Turkey; 7 Department of Public Health and Caring Sciences, Clinical Nutrition and Metabolism, Uppsala University, Uppsala, Sweden; 8 Servicio de Geriatría, Hospital Universitario Ramón y Cajal (IRYCIS), Ctra. Colmenar km 9.1, Madrid, 28034 Spain; 9 Institute for Evidence in Medicine, Medical Centre-University of Freiburg, Faculty of Medicine, University of Freiburg, Breisacher Str. 86, Freiburg, 79110 Germany; 10 Institute for Biomedicine of Aging, Friedrich-Alexander-Universität Erlangen-Nürnberg, Kobergerstr. 60, Nürnberg, 90408 Germany; 11 Department of Medicine, Kantonsspital Winterthur, Brauerstrasse 15, Postfach 834, Winterthur, Zurich 8401 Switzerland; 12 Department of Pediatrics, Paracelsus Medical University, Nuremberg, Germany

**Keywords:** Endpoint determination, Core Outcome Set, Aged, Malnutrition, Randomized controlled trials, Meta-analysis, Review, Delphi technique

## Abstract

**Background:**

Malnutrition (i.e., protein-energy malnutrition) in older adults has severe negative clinical consequences, emphasizing the need for effective treatments. Many, often small, randomized controlled trials (RCTs) testing the effectiveness of nutritional interventions for the treatment of malnutrition showed mixed results and a need for meta-analyses and data pooling has been expressed. However, evidence synthesis is hampered by the wide variety of outcomes and their method of assessment in previous RCTs. This paper describes the protocol for developing a Core Outcome Set (COS) for nutritional intervention studies in older adults with malnutrition and those at risk.

**Methods:**

The project consists of five phases. The first phase consists of a scoping review to identify frequently used outcomes in published RCTs and select additional patient-reported outcomes. The second phase includes a modified Delphi Survey involving experienced researchers and health care professionals working in the field of malnutrition in older adults, followed by the third phase consisting of a consensus meeting to discuss and agree what critical outcomes need to be included in the COS. The fourth phase will determine how each COS outcome should be measured based on a systematic literature review and a second consensus meeting. This will be followed by a dissemination and implementation phase. Patient and Public Involvement (PPI) representatives will contribute to study design, oversight, consensus, and dissemination.

**Conclusions:**

The result of this project is a COS that should be included in any RCT evaluating the effect of nutritional interventions in older adults with malnutrition and those at risk. This COS will facilitate comparison of RCT results, will increase efficient use of research resources and will reduce bias due to measurement of the outcome and publication bias. Ultimately, the COS will support clinical decision making by identifying the most effective approaches for treating and preventing malnutrition in older adults.

## Background

Many older adults are at high nutritional risk, with the prevalence of malnutrition (i.e. protein-energy malnutrition) ranging from 5 to 10% in community-dwelling older adults, 15–20% in older long-term care residents up to 20–30% in hospitalized older adults [1–3]. Malnutrition is associated with negative clinical outcomes, including functional decline [4], hospital re-admission and early death [5, 6], poor quality of life [7] and higher social and health care costs [8]. These severe consequences highlight the importance of effective treatment once malnutrition is diagnosed in older adults.

Unfortunately, many uncertainties remain regarding the effectiveness of nutritional interventions in older adults with malnutrition [9–13]. The randomized controlled trials (RCTs) included in previous reviews tested the effects of different interventions in different settings and in different populations and provide mixed results. Meta-analysis is a statistical procedure to increase statistical power when summarizing the evidence. There is a clear need for meta-analyses and for pooling individual participant data from RCTs to better understand which nutritional intervention works best in which setting and for which older adult [14]. Unfortunately, conducting these types of analyses is currently hampered by the fact that RCTs in this field use a large variety of outcomes to test the effects of the intervention and use a wide range of methods for assessing these outcomes.

A core outcome set (COS) is an agreed minimum set of outcomes that should be measured and reported in all clinical trials of a specific disease or trial population [15]. The increasing scientific interest in and importance of developing and using a COS in RCTs is shown by the steep rise in the number of peer-reviewed scientific publications on COS (from 26 in 2000 to 611 in 2021 as indicated in PubMed). Several initiatives importantly support the development and implementation of COS, such as the Core Outcome Measures in Effectiveness Trials (COMET [16]) and the Initiative and Outcome Measures in Rheumatoid Arthritis Clinical Trials (OMERACT [17]).

To enhance our knowledge on the effectiveness of nutritional interventions, there is a need to develop a COS that should be included in any future RCT evaluating a nutritional intervention in older adults with malnutrition and those at risk. The COS will be a minimum requirement and where necessary additional outcomes important for a specific research question can always be included. The inclusion of COS in all RCTs will not only facilitate comparison of results between RCTs and their combined evaluation, but will also increase efficient use of research resources by including those outcomes that are considered most relevant to malnutrition in older adults. Finally, inclusion of COS will reduce bias due to measurement of the outcome as outcome assessment will be more standardized as well as publication bias since the intervention effect on all COS outcomes should be reported. Ultimately, the COS will support clinical decision making by identifying the most effective approach for treating and preventing malnutrition in older adults.

This paper describes the research protocol for the development of a COS recommended for use in all future nutritional intervention trials in older adults with malnutrition and those at risk.

## Methods

This project was registered on the COMET Initiative registry of COS on October 22, 2021 [18] and was developed according to the COMET guidelines [19]. The reporting of this protocol follows the recommendation of the Core Outcome Set-STAndardised Protocol (COS-STAP) Items Statement [15].

### Project oversight

This project was initiated within the Special Interest Group Nutrition of the European Geriatric Medicine Society (EuGMS). An international Steering Group was formed among the SIG members to support the development of this COS. The Steering Group members, all authors of this protocol paper, have ample experience in conducting nutritional (intervention) research in older adults with malnutrition and those at risk, and/or the treatment of malnutrition in older adults in clinical practice. The project consists of five phases, described in detail below. Most members of the Steering Group were involved in performing the first phase of the project: the scoping review. For all other phases of the project a Core Group of three to four members of the Steering Group will be appointed to conduct the main work with advice and feedback on the process from the other Steering Group members.

### Scope

The COS will be developed to measure the efficacy and effectiveness of nutritional interventions that focus on increasing protein and/or energy intake in RCTs for older adults with malnutrition and older adults at risk of malnutrition.

### Project phases

**Fig. 1:**
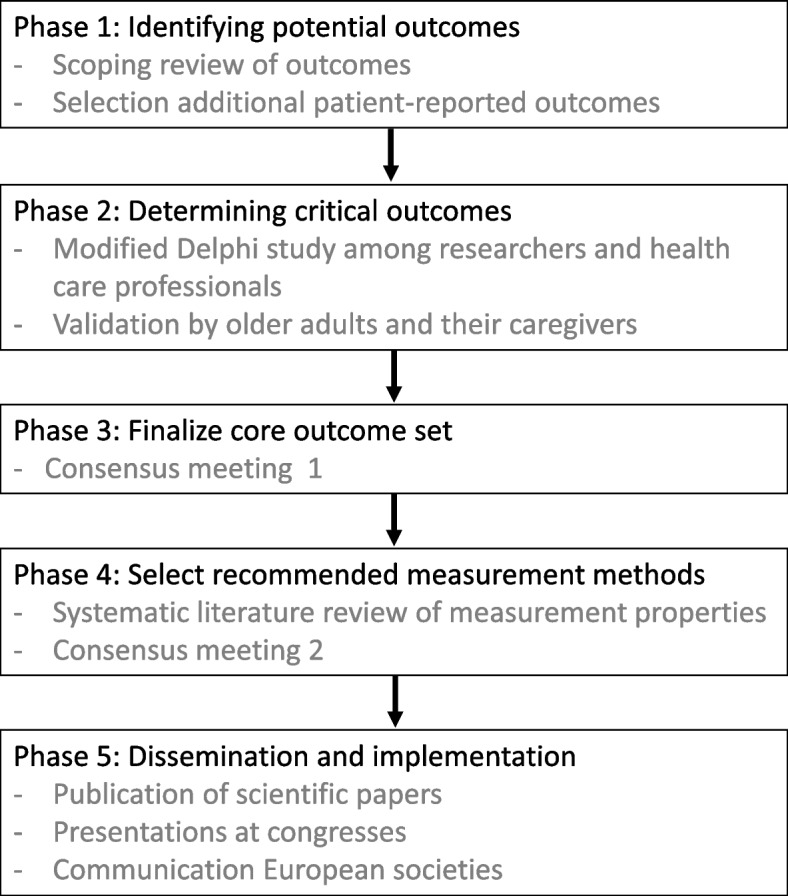
Overview of the project phases

The development of this COS will consist of five phases which are shown in Fig. 1.

#### Phase 1

A scoping review was performed in 2020–2021 to provide an overview of outcomes and their assessment methods used in nutritional intervention studies focused on the treatment of malnutrition in older adults. Details about the scoping review and the results can be obtained elsewhere [20]. Inclusion criteria used for the review are shown in Table 1. A total of 63 articles describing 60 RCTs were included in the review. All primary and secondary outcomes used as well as their frequencies of use and their assessment methods were reported for all 60 RCTs, as well as by setting (community, hospital and long-term care) [20].

**Table 1:** Inclusion criteria for the scoping review^a^

Domain	Specific criteria
Languages	All
Participants	Age 65 years and above. When the age range was not reported, a mean age of at least 70 years
	With malnutrition based on i) a screening/assessment capturing multiple aspects of undernutrition, or ii) BMI < 22 kg/m^2^, or iii) involuntary weight loss (as defined by study authors) *OR* at risk of malnutrition (based on a malnutrition screening tool)
	All health conditions
Settings	Community, hospital or long-term care/nursing home
Interventions	Nutritional intervention focused on increasing the intake of protein and/or energy
Control condition	The contrast between the randomized groups is the increase in protein and/or energy intake
Study design	RCT, including quasi-randomized, cluster-randomized and randomized cross-over design
Publication type	Result paper, protocol paper, trial registration
Outcomes	All

^a^For details, please see [20]

#### Phase 2

Phase 2 will consist of a modified Delphi study. An international, online Delphi survey will be conducted in English, aiming to reach consensus on core outcomes (per setting when needed).

##### Stakeholders

Stakeholders will include 1) researchers with experience in performing studies in older adults with malnutrition and/or those at risk, including those working for industry, 2) health care professionals (HCPs), including physicians (i.e., geriatricians, general practitioners, internists), registered dieticians, and other allied health professionals such as nurses, with experience in the treatment of older adults with malnutrition and those at risk, and 3) older adults with malnutrition or at risk of malnutrition, older adults who experienced malnutrition in the past 2 years, and caregivers of older people with malnutrition or at risk of malnutrition. The latter stakeholder group will be involved as Patient and Public Involvement (PPI) representatives who will contribute to the design and oversight of the study, will provide feedback on outcomes, and will also take active part in meetings including the consensus meeting. As we aim to recruit international experts in the field of malnutrition in older adults, we will also recruit experts who have important public health advisory roles regarding (mal)nutrition and geriatrics. For example, several experts as well as members of the Steering Group play important roles in national and European societies through which they are involved in the development of preventive strategies and treatment guidelines, and many have an advisory role in national policy making regarding services and programs regarding nutrition and geriatrics*.*

##### Delphi participants and recruitment strategy

The Delphi survey will be conducted among researchers and HCPs. We will follow a 2-tier approach. More specifically, we will first recruit potential stakeholders by addressing invitations to members of scientific societies (e.g., EuGMS and the European Society for Clinical Nutrition and Metabolism (ESPEN)) or other stakeholders from our networks with known research and/or clinical expertise in the area, working in each of the three settings community, hospital, and long-term care. To increase the involvement of expert stakeholders outside of Europe, we will identify researchers who are first or last author of three or more international, peer-reviewed scientific publications on the topic of malnutrition in older persons. Identified experts will also be asked to nominate local HCPs with known clinical expertise in the field of malnutrition in the respective setting in the final section of the survey. Thus, we will ensure that the stakeholders have the necessary relevant expertise in this field and that inclusion of professionals from different settings and with different backgrounds is as equally distributed as possible.

If this strategy does not lead to a sufficient number of participants (see below), then we will open our recruitment more widely by addressing invitations to HCPs via professional registration bodies, national health service newsletters at a local level, including hospital trusts, community dietetic services, voluntary/third sector, and primary care organisations (e.g. Clinical Commissioning Groups or equivalent). We will also target our recruitment to organisations caring for older people living in the community (such as Frailty Multi-Disciplinary Teams or mobile geriatric assessment units), and for those living in residential care, targeting HCPs who provide nutritional care for nursing home residents.

##### Sampling

We will implement a purposive sampling strategy to have a balanced representation across three main settings: 1) hospital, 2) community, and 3) residential care/nursing home. We aim to recruit researchers and HCPs at a 50/50 ratio to achieve a balanced representation between clinical practice and academia. We will aim for a balanced representation by gender and geographical location (country of professional practice) of the individuals participating in the Delphi survey across the two included stakeholder groups (researchers and HCPs). To allow for attrition we aim to recruit 200 participants in total, in order to increase the likelihood of having complete Delphi rounds for 50 participants per setting (community, hospital, and long-term care).

##### Patient and public involvement (PPI)

To ensure PPI, we already have involved patients and members of the public in the development of this protocol. Two members of the public from the UK and one from Turkey, representing older people and/or caregivers of older people, were asked to provide feedback on the list of outcomes to be included in the Delphi survey, including patient-reported outcomes, and reviewed a summary of the protocol.

To further ensure PPI, we will also involve members of the public, including older people who have lived experience of being underweight or being managed for malnutrition, informal caregivers (family and friends) and paid caregivers (employed by home care agencies, privately, or by nursing homes), who will be asked to provide feedback on the draft COS after the completion of all Delphi rounds. PPI members will be recruited via patient and caregiver associations, charity organisations at a local and national level, and co-investigators’ existing connections and networking from other PPI activities/previous projects. This feedback will be obtained in the local language to avoid any language barriers. Two PPI representatives will be invited to the Steering Group meetings and will take part in the voting process at the consensus meeting.

##### Data management and confidentiality

Information on the study and invitations to participate will be sent to publicly available email addresses. From participants consenting to participate an email address will be stored together with their responses during the data collection phase, which will allow approaching participants for the second Delphi round and investigating attrition (see below). The final list of participants will remain confidential to all except for the Core Group performing the Delphi Study. After completing data collection, participants will be allocated a unique identifier to anonymise their responses. The collected data will be stored password protected on a university data server. Ethical approval for conducting the Delphi survey was obtained from the Universidade Nova de Lisboa medical school, Lisbon, Portugal (No. 86/2022/CEFCM).

##### Delphi rounds and consensus procedure

Participants will be asked to commit to completion of two rounds of the Delphi Study. Rounds will be open for a 3-week period, and a reminder email will be sent on day 14 to participants yet to complete their survey. If required, additional strategies such as extending the survey deadline and personalised reminders may be used to increase response rates. The time between the end of the first round and the start of the second round will not exceed 4 weeks to allow sufficient time for data analysis and preparing the next round, while lowering the risk of participant attrition.

In round 1, participants will be asked to indicate in which setting (community, hospital and long-term care) they predominantly work and will be asked to complete the Delphi for that setting only. This approach will allow deriving core outcomes per setting when necessary, as the results of the scoping review indicated that the set of most frequently used outcomes differed by setting and suggested that some outcomes used in one setting were (almost) never used in another setting [20]. As the outcome follow-up time in RCTs may influence the rating of outcomes [19], participants will be asked to indicate which outcome follow-up time (short-term (≤12 weeks) or long-term (> 12 weeks)) of nutritional interventions they have most experience with or consider most relevant, and will be asked to complete the Delphi Study with that follow-up time in mind. Participants will also be asked to indicate their gender, country where they work, and background/discipline to investigate the extent to which the representation is balanced.

Round 1 will include the outcomes obtained in the first phase of the project (scoping review). The Steering Group will review the list of outcomes obtained from the scoping review and will (re)group outcomes into outcome domains when necessary to improve clarity. Furthermore, the Steering Group will pre-select a group of outcomes from the scoping review that are considered not critical for inclusion in the Delphi and therefore should not be rated, using an agreement of 80% or more. The decision to exclude these outcomes will also be verified in round 1 of the Delphi (see below). In addition, a set of Patient Reported Outcomes (PROs) not yet identified in the scoping review and considered relevant for the scope of this COS by the Steering Group (using an agreement of 80% or more) or by the PPI participants will be included in round 1. This set of additional PROs will be derived from the Patient-Reported Outcomes Measurement Information System® (PROMIS®) to ensure the inclusion of validated and psychometrically sound measures [21]. These PROs will be added to purposely increase the likelihood that outcomes potentially considered relevant by older adults will be included in the final core outcomes of the Delphi Study.

Each outcome will be accompanied by a clear description to facilitate common understanding of the construct. Participants will be instructed to rate the importance of each outcome on a Likert scale from 1 to 9, with 1 to 3 labelled ‘not important’, 4 to 6 ‘important but not critical’, 7 to 9 ‘critical’ [22]. A text box will be provided for participants to motivate their rating and to make any other comments (for example on combining or renaming outcomes, or not being familiar with the outcome). The order of the domains will be randomised. Also, the group of pre-selected outcomes from the scoping review considered not critical for COS inclusion by the Steering Group will be shown and participants will be asked to agree or disagree with the exclusion of these outcomes and to motivate their choice in a text box. Finally, the participants will be asked to list additional outcomes that they believe are critical and have not been mentioned.

In round 2 the number of outcomes to be rated will be reduced in order to lower participant burden and attrition. Outcomes will be grouped as set of outcomes to be included in the COS when, potentially within a setting and follow-up time, 75% of the participants score the outcome as ‘critical’ and less than 15% of the participants score the outcome as ‘not important’. Similarly, outcomes will be grouped as a set of outcomes *not* to be included in the COS when over 75% of the participants score the outcome as ‘not important’ and less than 15% of the participants score the outcome as ‘critical’. To purposely increase the likelihood that outcomes potentially considered relevant by older adults will be included in the core outcomes, less strict consensus criteria will be applied for the PROs. PROs will be included in COS when over 60% of the participants score the outcome as ‘critical’ and less than 15% of the participants score the outcome as ‘not important’, and will be excluded when over 60% of the participants score the outcome as ‘not important’ and less than 15% of the participants score the outcome as ‘critical’. Participants will be asked to agree or disagree with the groups of included and excluded outcomes and to motivate their choice in a text box. Outcomes for which no consensus was obtained in round 1, will be (re)rated by the participants using a Likert scale from 1 to 9. The order of the domains and outcomes will be randomised. At the round 2 survey, participants will receive a summary of their own responses for round 1 as well as the distribution of scores from all participants of round 1. Outcomes newly proposed in round 1 will be reviewed by the Steering Group and added for rating in round 2 when deemed necessary with an agreement of 80% or more. The ratings for round 2 will be analysed similar to round 1. Participants who fully complete the two rounds will be asked to express their interest in participating in two consensus meetings.

Missing data will be minimized using the online survey development software options, for example by not allowing to (accidentally) skip questions. Potential bias arising from participant attrition, for example between round 1 and round 2, will be assessed by examining the differences in round 1 scores for each outcome among those who do and do not complete round 2 [19].

#### Phase 3

An online consensus meeting will be organized with 10–15 persons selected from those who expressed their interest, balanced according to stakeholder group, setting, gender, country and background/discipline, and members of the Steering Group, including two PPI representatives. The outcomes to be included in the COS, outcomes not to be included in the COS, and outcomes for which no consensus could be derived based on the Delphi Study among researchers and HCPs will be presented - for each setting when necessary. The summarized results obtained from the PPI will also be presented. After discussing these results and the motivations for in- or exclusion, and after discussing how to incorporate the views of the older adults in the COS, participants will be asked to anonymously vote each outcome as ‘yes’ or ‘no’ for inclusion in the final COS. When 70% or more of participants rated ‘yes’, an outcome will be included in the final COS – for each setting when necessary.

#### Phase 4

While phases 1–3 will establish *which* outcome should be included in the COS, phase 4 will establish *how* the outcomes included in the COS should be measured. For the outcomes included in COS, the assessment methods identified in the scoping review of phase 1 will be evaluated by a Core Group according to their measurement properties [23]. The Steering Group will be allowed to add relevant assessment methods not listed in the scoping review and to add any newly developed assessment methods not yet published in publications. The following nine measurement properties used by the COnsensus-based Standards for the selection of health status Measurement INstruments (COSMIN) initiative will be evaluated as far as available: internal consistency, reliability, measurement error, content validity, structural validity, hypotheses testing, cross-cultural validity, criterion validity, and responsiveness [24]. The obtained results will be presented and discussed during a second virtual consensus meeting. Other important aspects, including feasibility, practicality, economical aspects and equitable access, will also be considered. The preferred assessment method will be determined (by setting when necessary) by voting, using the same criterion as used in phase 3.

#### Phase 5

In phase 5 the results of the project will be disseminated and implemented. The Steering Group members will actively disseminate the final COS to the scientific community and HCPs and support implementation of COS via their own professional networks as well as through their memberships and roles in international societies and scientific journals. Representatives from (local) governments and health policy makers will be informed about our project and will be involved in the implementation. Results of this project will be published in international, peer-reviewed scientific journals and presented at the annual meetings of EuGMS and ESPEN by organizing a dedicated symposium and by submitting individual posters and oral presentations. PPI members will contribute to the dissemination of the final results to a wider audience via their connections with patient and caregiver associations.

### Limitations

A limitation of this study is that we have not invited older adults with malnutrition and those at risk to take part in the Delphi online survey. We were concerned that older adults with malnutrition in the hospital or long-term care setting often suffer from acute disease and/or severe functional or cognitive limitations that would prevent completing the online survey. Language barriers completing the English survey also played a role, as we were unfortunately limited in our resources and therefore could not undertake a translation and validation of the survey in different languages. We therefore decided to adopt a different strategy and involve older people and caregiver representatives as PPI, in order to obtain and incorporate their views in developing the protocol and the Delphi survey rounds, as well as give them the right to vote at the consensus meeting.

Another potential limitation is that although the majority of malnutrition intervention trials have been conducted in high-income countries [20], there are many older populations globally that are affected by health disparities, for example due to living in a low-income country, rural areas or belonging to an ethnic minority. Available funding resources can vary considerably amongst different countries and settings, affecting research processes and ultimately the implementation of interventions at a wider scale. Therefore, the COS that will result from the current study needs to be then considered for its feasibility and applicability in terms of socioeconomic context and health equity. This will be included in the discussion during the consensus meeting.

## Data Availability

Data sharing is not applicable to this article as datasets were not yet generated.

## Abbreviations

BMI: Body mass index
COMET: Core Outcome Measures in Effectiveness Trials
COS: Core Outcome Set
COSMIN: COnsensus-based Standards for the selection of health status Measurement INstruments
COS-STAP: Core Outcome Set-STAndardised Protocol
ESPEN: European Society for Clinical Nutrition and Metabolism
EuGMS: European Geriatric Medicine Society
HCP: Health care professional
PPI: Patient and public involvement
PRO: Patient Reported Outcome
PROMIS®: Patient-Reported Outcomes Measurement Information System®
RCT: Randomised controlled trial
